# Scientific

**Published:** 1889-09

**Authors:** 


### SCIENTIFIC ITEMS.

**Discovery of an Assyrian Library Three Thousand Five Hundred Years Old.**—The Victoria Institute held its meeting before a large audience, at Adelphi Terrace, July 1st. Capt. Francis Petrie, the honorary secretary, read the annual report, which showed that there were now more than 1300 members of the Institute. A most interesting paper by Prof. Sayce was the feature of the occasion. It was chiefly an account of certain Babylonian discoveries that have lately been made, which show that fifteen centuries before our era there was an active literary intercourse going on throughout the civilized world of Western Asia, chiefly by means of the Babylonian language and script. Great libraries were collected formed of clay tablets, with cuneiform inscriptions. The archives discovered treat mainly of the conquests of Amenophis III., also throwing much light on the contemporaneous history of adjacent countries. Prof. Sayce hopes that there may yet be found in the sands of Syria and Palestine more of these treasures of such priceless value to our knowledge of those times.

A correspondent of the *Lancet* gives the following method of self-asphyxiation as an effectual remedy for insomnia in his own case:

"After taking a deep inspiration he holds his breath till discomfort is felt; then repeats the process a second and third time. As a rule, this is enough to procure sleep. A slight degree of asphyxia is thus relied on as a soporific agent."

A Swedish scientist claims to have discovered the secret of artificially petrifying wood, by which means he believes edifices may be built of wood and converted into stone. At present the cost is about $500 per cubic inch, so that the discovery does not promise an immediate revolution in building.

**Cave Dwellers Found in Mexico.**—A dispatch from Deming, New Mexico, says: "Leutenant Schwatka has arrived here. His party has been successful beyond expectations in their explorations, and especially in Southern Chihuahua, where living cliff and cave dwellers were found in great abundance, wild as any of the Mexican tribes at the time of Cortez's conquest. The abodes they live in are exactly similar to the old, abandoned cliff dwellings of Arizona and New Mexico, about which there has been so much speculation. It was almost impossible to get near them, so wild and timid were they. Upon the approach of white people, they flee to their caves by notched sticks placed against the face of the cliffs, if steep, although they can ascend vertical stone faces if there are the slightest crevices for their fingers and toes.

"These cliff dwellers are sun worshipers, putting their new-born children out in the full rays of the sun the first day of their lives, and showing many other forms of devotion to the great luminary. They are usually tall, lean, and well-formed, their skin being a blackish red, much nearer the color of the negro than the copper-colored Indian of the United States.

"Schwatka claims that nothing has heretofore been known about these people, except by the half-Indian mountain Mexicans, and thinks his investigation will be of immense anthropological and archæological value. He estimates the cave and cliff dwellors to be from 3,000 to 12,000 in number, armed only with bows, arrows, and stone hatchets."—*Scientific American.*

Iron and steel are now usually distinguished by the use of aqua fortis, which, when applied to a surface of steel, produces a black spot. On iorn it has no effect, leaving the metal perfectly clean. By this test the slightest vein of iron in steel can readily be detected.

**Photography Applied to the Prediction of the Weather.**—With regard to the accident which has occurred to the German navy at Apia, it might be advisable to refer once more to the theory of Dr. Zenger, of Prague, who suggested, as it will be remembered, to make use of photography for the prediction of the weather. According to the doctor, photographs of the sun taken on orthochromatic plates offer a most infallible means to indicate with almost absolute certainty the approaching atmospheric and subterranean disturbances at least twenty-four hours before their setting in. In these photographs zones are often to be seen around the sun's disk—*i. e.*, rings of circular or elliptical form, of white or grayish color—and if these zones appear of very large diameter, and of unusual heaviness, this indicates that violent storms, thunderstorms, or magnetical disturbances will soon set in at the place of observation. At every ship's station should therefore be established a small photographic laboratory, in which photographs of the sun could be taken as often as possible. A much more reliable prediction of the weather would be afforded by this means than by the aid of the barometer now generally in use for this purpose, and precautions could therefore be taken in good time.—*H. E. Gunthur, in Phoco. News.*

**Meteorites.**—From an exhaustive study of the large collection of meteorites at Harvard College, the conclusion has been arrived at, that many of the masses of meteoric iron now known are cleavage crystals, broken off, probably, by the impact of the mass against the atmosphere. It is found that these masses show cleavings parallel to the planes of all the three fundamental forms of the isometric or rugular system. From all that appears, the theory has come to be entertained that the masses of meteorites were thrown off from a sun among the fixed stars, and that they were slowly cooled while revolving in a zone of intense heat.—*Pop. Science News.*

